# A Comparative Study on the Performance of GSCA and CSA in Parameter Recovery for Structural Equation Models With Ordinal Observed Variables

**DOI:** 10.3389/fpsyg.2018.02461

**Published:** 2018-12-05

**Authors:** Kwanghee Jung, Pavel Panko, Jaehoon Lee, Heungsun Hwang

**Affiliations:** ^1^Educational Psychology and Leadership, Texas Tech University, Lubbock, TX, United States; ^2^Psychology, McGill University, Montreal, QC, Canada

**Keywords:** generalized structured component analysis, alternating least squares estimation, maximum likelihood estimation, diagonally weighted least squares estimation, structural equation modeling, covariance structure analysis, monte carlo simulation

## Abstract

A simulation based comparative study was designed to compare two alternative approaches to structural equation modeling—generalized structured component analysis (GSCA) with the alternating least squares (ALS) estimator vs. covariance structure analysis (CSA) with the maximum likelihood (ML) estimator or the weighted least squares mean and variance adjusted (WLSMV) estimator—in terms of parameter recovery with ordinal observed variables. The simulated conditions included the number of response categories in observed variables, distribution of ordinal observed variables, sample size, and model misspecification. The simulation outcomes focused on average root mean square error (RMSE) and average relative bias (RB) in parameter estimates. The results indicated that, by and large, GSCA-ALS recovered structural path coefficients more accurately than CSA-ML and CSA-WLSMV in either a correctly or incorrectly specified model, regardless of the number of response categories, observed variable distribution, and sample size. In terms of loadings, CSA-WLSMV outperformed GSCA-ALS and CSA-ML in almost all conditions. Implications and limitations of the current findings are discussed, as well as suggestions for future research.

Latent variable modeling has become a methodological mainstay in social and behavioral sciences research and beyond. Specifically, structural equation modeling (SEM; Jöreskog, 1970, 1973), a method of path analysis using latent variables, has been extensively utilized and evaluated by substantive experts and methodological researchers. However, as there are many different approaches for conducting SEM (e.g., covariance structure analysis, partial least squares path modeling, generalized structured component analysis), many questions regarding optimal modeling approaches still exist. For instance, responding to estimation challenges in the context of ordinal observed variables and developing strategies to counteract model misspecifications are areas of importance in SEM research (e.g., Flora and Curran, 2004; Bandalos, 2008; Li, 2016).

Recently, Li (2016) carried out a simulation study to examine the performance of covariance structural analysis (CSA) with ordinal observed variables, comparing the conventional normal-theory estimation method, maximum likelihood (ML), with the methods employing a robust correction for non-normality. ML estimation uses a sample covariance matrix as input data under the assumption of continuous observed variables and multivariate normality of the observed variables, while the robust methods, diagonally weighted least squares (DWLS) estimation and unweighted least squares (ULS) estimation, use a polychoric correlation matrix with the assumption that a continuous and normally distributed latent variable underlies each observed variable. In this simulation, the study conditions were manipulated by varying distributional properties of ordinal observed variables, the number of response categories, and sample size. The simulation results revealed that compared to ML, DWLS, and ULS produced more accurate estimates for the factor loadings in all differing number of response categories; as well as for the path coefficients in nearly all asymmetric distribution conditions. Similar to previous findings (see Muthén et al., 1997), both DWLS and ULS did not require a large sample for the parameter recovery—i.e., a sample size of 200 or 300 would be sufficient for accurate parameter estimation. In short, Li (2016) showed that when observed variables in SEM are ordinal variables, DWLS and ULS are favorable to ML in terms of parameter recovery. Nevertheless, ML would be recommended for cases with observed variables that have symmetric distributions in a large sample.

In the current study, we incorporate generalized structured component analysis (GSCA; Hwang and Takane, 2004, 2014), which is component-based SEM and an alternative to factor-based CSA (Tenenhaus, 2008; Rigdon, 2012), for use with continuous and ordinal observed variables. GSCA postulates that weighted composites or components of indicators may serve as proxies for latent variables as in principal component analysis, while factor-based CSA assumes that common factors may approximate latent variables as in common factor analysis. As such, GSCA is capable of providing unique latent variable scores while avoiding latent variable score indeterminacy in CSA. More importantly, GSCA can accommodate models with a higher degree of complexity, which may be difficult or impossible for factor-based SEM due to technical difficulties such as model non-identification, presence of improper solution, non-convergence, and so on. Hwang and Takane (2014) highlighted the practical usefulness of GSCA in regard to flexibility in model specification and computational efficiency in parameter estimation. At a glance, GSCA uses an alternating least squares (ALS; De Leeuw et al., 1976) algorithm for parameter estimation and employs the bootstrap method (Efron, 1982) to assess the reliability of parameter estimates without rigid distributional assumptions (e.g., multivariate normality assumption that is often made in CSA). Thus, it allows for stable parameter estimates even in a small sample.

A simulation study by Hwang et al. (2010) examined the parameter recovery in GSCA-ALS and CSA-ML using a relatively simple model with three latent variables and three normally distributed, continuous observed variables per latent variable. This study showed that when the model is correctly specified, CSA-ML tends to produce better parameter estimates compared to GSCA-ALS. In contrast, when the model is misspecified, GSCA-ALS tends to have superior parameter recovery. These findings suggest that GSCA-ALS is recommended over CSA-ML unless a correct model specification is ensured. Unfortunately, little is known for the ordinal variable case, even though Likert-type ordinal scales are very commonly used to operationalize latent variables in applied research. If researchers inadvertently treat ordinal variables as continuous, it may lead to unreliable parameter estimates and incorrect inferences (Flora and Curran, 2004). Therefore, a thorough, empirical examination is imperative to understand the performance of GSCA-ALS, in comparison with CSA, on parameter recovery for ordinal observed variables.

The organization of this article is as follows. The following sections demonstrate GSCA and CSA approaches to SEM and discuss some estimation issues relevant to those methods. Then, the design and analysis procedure of a Monte Carlo simulation study are presented. In the final section, the authors discuss study findings and implications, as well as limitations and directions for future research. The present study may contribute to the literature by allowing for researchers and practitioners to acknowledge viable options and potential consequences and implications of their choice when conducting a SEM analysis with ordinal observed variables.

## Generalized Structured Component Analysis with Alternating Least Squares (GSCA-ALS)

Generalized structured component analysis (GSCA) is component-based SEM (Tenenhaus, 2008). GSCA defines latent variables as weighted composites or components of observed variables as follows:

$$\gamma_{i}=Wz_{i}$$

where *z*_*i*_ denotes a vector of observed variables for a respondent *i* (*i* = 1, …, *N*), γ_*i*_ is a vector of latent variables for a respondent *i*, and *W* is a matrix consisting of component weights assigned to observed variables. This equation is called the weighted relation model. Both *z*_*i*_ and γ_*i*_ are assumed to be standardized with zero mean and unit variance. Here, components or weighted composites of the indicators are assumed to be proxies for latent variables in GSCA or other multivariate methods, aiming to capture the most representative variation in the indicators. In contrast, in factor-based CSA, common factors are postulated as latent variables, which are assumed to only account for covariances among indicators.

GSCA comprises two additional equations for model specifications. One is for the measurement model which specifies the relationships between observed variables and latent variables, and the other is for the structural model which captures the relationships among latent variables. The measurement model is given by the following:

$$z_{i}=C\gamma_{i}+\varepsilon_{i},$$

where *C* is a matrix of loadings relating latent variables to observed variables and ε_*i*_ is a vector of residuals for *z*_*i*_. The structural model is defined by the following:

$$\gamma_{i}=B\gamma_{i}+\xi_{i},$$

where *B* is a matrix of path coefficients connecting latent variables among themselves and ξ_*i*_ is a vector of residuals for γ_*i*_.

Then, the GSCA model is derived from integrating the three submodels into a single, general model as follows:

$$\begin{array}{l} \;\left[\begin{array}{c} z_{i}\\ \gamma_{i} \end{array}\right]=\left[\begin{array}{c} C\\ B \end{array}\right]\gamma_{i}+\left[\begin{array}{c} \varepsilon_{i}\\ \xi_{i} \end{array}\right]\\ \left[\begin{array}{c} I\\ W \end{array}\right]z_{i}=\left[\begin{array}{c} C\\ B \end{array}\right]Wz_{i}+\left[\begin{array}{c} \varepsilon_{i}\\ \xi_{i} \end{array}\right]\\ \;Vz_{i}=AWz_{i}+e_{i}, \end{array}$$

where

$$V=\left[\begin{array}{c} I\\ W \end{array}\right],\;A=\left[\begin{array}{c} C\\ B \end{array}\right],\;e_{i}=\left[\begin{array}{c} \varepsilon_{i}\\ \xi_{i} \end{array}\right],$$

and *I* is an identity matrix (Hwang and Takane, 2004, 2014).

The unknown parameters of GSCA (*W* and *A*) are estimated such that the sum of squares of all residuals (*e*_*i*_) is as small as possible across all respondents. This pertains to minimizing the following least squares criterion:

$$\phi =\sum_{i=1}^{N}{\left(Vz_{i}-AWz_{i}\right)}^{\prime }\left(Vz_{i}-AWz_{i}\right),$$

with respect to *W* and *A*, subject to the constraint that each latent variable is standardized, $\sum_{i=1}^{N}\gamma_{id}^{2}=N$, where γ_*id*_ is the *d*th element of γ_*i*_. An ALS algorithm was developed to minimize the least squares criterion—we therefore refer to this approach as GSCA-ALS. This algorithm alternates two main steps as many times as necessary until all parameter estimates stabilize. In the first step, for fixed *W*, *A* is updated in the least squares manner. In the second step, *W* is updated in the least squares sense for fixed *A* (for a detailed description of the algorithm, see (Hwang and Takane, 2014)). In GSCA-ALS, a bootstrap method is utilized to calculate the standard errors and confidence intervals of parameter estimates without the multivariate normality assumption of observed variables. The bootstrapped standard errors or confidence intervals can be used for testing the statistical significance of the parameter estimates.

The simulation study by Hwang et al. (2010) investigated the performance of GSCA-ALS and CSA-ML using a simple model with three latent variables and normally distributed, continuous observed variables. They arrived at two major conclusions. First, when the model is correctly specified, factor-based CSA-ML may be used over GSCA-ALS. Second, when the model is incorrectly specified, GSCA-ALS may be chosen over factor-based CSA-ML. In another Monte Carlo simulation study, Dynamic GSCA (Jung et al., 2012), an extended model of GSCA for longitudinal and time series analysis, showed reasonable parameter recovery rates with a very complex model (i.e., seven latent variables were nearly fully connected by contemporaneous reciprocal relations and by autoregressive paths), even in small samples (i.e., *n* = 50 or 100).

## Covariance Structural Analysis with Maximum Likelihood (CSA-ML)

Using the notations commonly used in covariance structure analysis (CSA) (Bollen, 1989; Kaplan, 2008), the measurement models for endogenous variables and exogenous variables are defined by the following:

$$y=\Lambda_{Y}\eta +e$$

and

$$x=\Lambda_{x}\xi +\delta ,$$

where *y* is a vector of endogenous observed variables, η is a vector of endogenous latent variables, x is a vector of exogenous observed variables, ξ is a vector of exogenous latent variables, Λ_*Y*_ and Λ_*x*_ are factor loading matrices, and *e* and δ are uniqueness vectors, respectively. The structural model is defined by the following:

η=Bη+Γξ+ζ

where *B* is a matrix of path coefficients relating the latent endogenous variables to each other, Γ is a matrix of path coefficients relating endogenous variables to exogenous variables, and ζ is a vector of disturbance terms.

Assuming the multivariate normality of observed variables, the ML estimator produces parameter estimates that maximize the fit function *F*_*ML*_ (Bollen, 1989):

$$F_{ML}=ln\left|\sum \left(\theta \right)\right|+trace\left[S\Sigma^{-1}\left(\theta \right)\right]-ln\left|S\right|-r$$

where θ denotes a vector of model parameters, ∑(θ) is a model implied covariance matrix, *S* is a sample covariance matrix, and *r* is the total number of observed variables.

CSA-ML assumes the observed data to be multivariate normally distributed, but often this assumption is not tenable for ordinal variables. In such case, CSA-ML can yield remarkably erroneous parameter estimates (Boomsma, 1982; Chou et al., 1991). This problem is exacerbated with small samples—for instance, Anderson and Gerbing (1984) and (Boomsma, 1982, 1985) showed that the chance of an improper or inadmissible solution, such as negative residual variance estimates, increases with non-normally distributed ordinal observations from a small sample.

## Covariance Structural Analysis with Weighted Least Squares (CSA-WLSMV)

Weighted least squares (WLS) is an asymptotically distribution-free estimator for non-normal continuous or categorical data (Brown, 2006). It utilizes a consistent estimate of the asymptotic covariance matrix of sample variances and covariances (Browne, 1984). Muthén (1984) adapted this approach for SEM with ordinal observed variables by making the assumption that a normal, latent response distribution underlies each ordinal observed variable in the population. That is, first, thresholds are estimated from the univariate marginal distribution, and then polychoric correlations are estimated from the bivariate marginal distributions for the given threshold estimates (Olsson, 1979). A consistent estimator of the asymptotic covariance matrix of the polychoric correlation and threshold estimates is used as a weight matrix Ω to obtain parameter estimates by minimizing the WLS fit function *F*_*WLS*_ (Muthén, 1984):

$$F_{WLS}={\left[s-\sigma (\dot{\theta })\right]}^{\prime }\Omega^{-1}\left[s-\sigma (\dot{\theta })\right]$$

where *s* is a vector containing the non-duplicated, vectorized elements of sample statistics (i.e., polychoric correlation and threshold estimates), $\dot{\theta }$ is a vector of model parameters, is a model-implied vector consisting the non-dupulicated, vectorized elements of the polychoric correlation matrix $\Sigma^{*}\dot{\theta }$ [i.e., $\sigma \dot{\theta }=vec\left(\Sigma^{*}\dot{\theta }\right)$].

Previous simulations studies have shown that WLS is prone to non-covergence problems with small samples and complex models (Flora and Curran, 2004). When sample sizes are small, the estimated asymptotic covariance matrix shows great sampling variation, and its inversion is typically infeasible. Moreover, as the number of ordinal observed variables increases, the size and invertibility of the weight matrix grow rapidly, leading to computational challenges and numerical issues in parameter estimation (Browne, 1984). To circumvent these problems, diagonally weighted least squares (DWLS, Christoffersson, 1977) estimation has been proposed by choosing a reduced and invertible asymptotic covariance matrix. The fit function can be represented as:

$$F_{DWLS}={\left[s-\sigma (\dot{\theta })\right]}^{\prime }\Omega_{D}{\;}^{-1}\left[s-\sigma (\dot{\theta })\right]$$

where Ω_*D*_ involves only diagonal elements of the estimated asymptotic covariance matrix of the polychoric correlation and threshold estimates. The weighted least squares mean and variance adjusted (WLSMV) estimator (Asparouhov and Muthén, 2010) for DWLS estimation was chosen for the current investigation. WLSMV approximates the mean of the expected χ^2^ distribution as well as its variance. WLSMV has been found to outperform WLS in case of small samples and complex models, even when non-normally-distributed ordinal data with small number of categories were analyzed (Flora and Curran, 2004; Bandalos, 2008; DiStefano and Morgan, 2014).

## Materials and Methods

The relative performance of CSA (using the ML or WLSMV estimator) and GSCA (using the ALS estimator) with non-normally-distributed ordinal data was analyzed using a Monte Carlo simulation. Considering a comparability with previous simulation work, a similar five-factor data-generating model as used in Li (2016) was utilized to compare primarily the parameter recovery under the conditions of correct or incorrect model specification. Additionally, the current simulation considered several condition factors such as different number of response categories, level of distributional asymmetry, and sample size.

### Population Model

**Figure 1:**
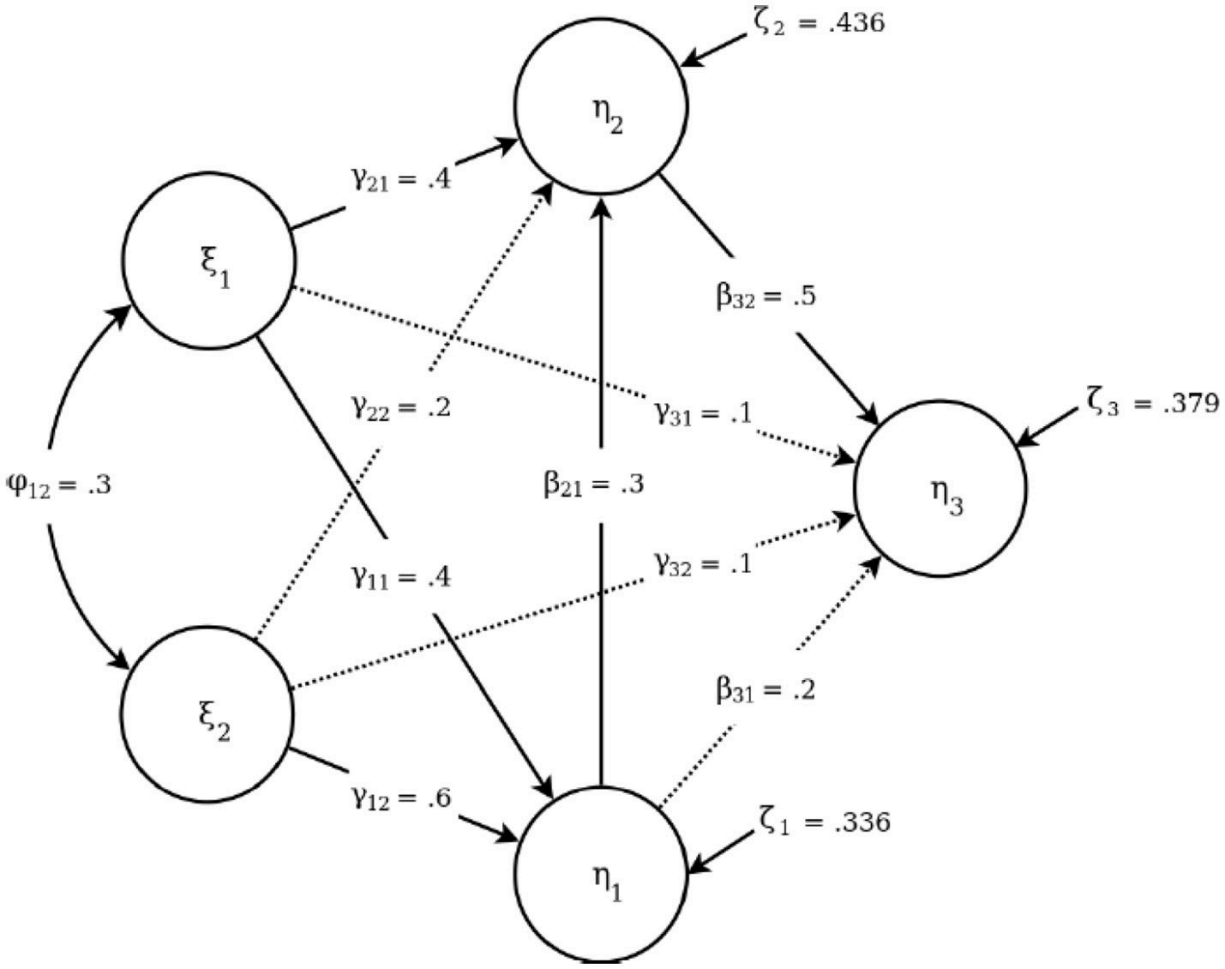
The population structural model with five latent variables. The paths omitted in the misspecification condition are displayed as dotted lines.

Figure 1 depicts the structural layout of the data-generating model (i.e., population model) used for the current simulation. As in the study by Li (2016), a five-factor structural equation model with four ordinal observed variables were selected to ensure a representative structural equation model from an applied standpoint. The structural part of the population model (i.e., structural model) contains three endogenous and two exogenous latent variables with a series of structural regression paths specified between them, which range in magnitude from a coefficient of 0.1 to 0.6. As shown in Figure 1, the six endogenous-to-exogenous paths are labeled by γ; and the three exogenous-to-exogenous paths are labeled by β. The single correlation term between the two endogenous latent variables is labeled by ϕ. Additionally, the measurement part of the population model (i.e., measurement model) was set to be homogenous between all the latent variables, i.e., each latent variable featured four observed variables with standardized loadings of 0.8, 0.7, 0.6, and 0.5.

### Simulation Design

The population model was estimated with either correct or incorrect structural model specification. Under structural misspecification, we have chosen to omit the four weakest paths in the model, namely, γ_22_, γ_31_, γ_32_, and β_31_. This choice represents the *a priori* decision to exclude effects that have been shown to be non-significant in previous studies. The exclusion of such paths is common in SEM; it presents a seemingly acceptable detraction from an unknown data-generating model based on a negligible difference in model fit. Those three omitted paths under model misspecification are depicted as dotted lines in Figure 1.

For observed variables, we simulated several different instances of ordinal data configuration that are most commonly encountered in applied research. Specifically, we manipulated the number of response categories by using observed variables with 4, 5, 6, and 7 categories. In addition, we considered two different distributions for those observed variables; a symmetric distribution with zero skewness and kurtosis ranging from −0.49 to −0.48, and an asymmetric distribution with skewness from −1.39 to −1.38 and kurtosis from 1.14 to 1.19.

Sample size was also one of the condition factors under study. In the CSA literature, there are many articles focusing on finding an optimal sample size within different data environments, with recommendations usually varying by the number of parameters in the model. On the other hand, GSCA models make no such restrictions, and are reportedly able to provide adequate estimates at sample sizes commonly considered small under CSA (Hwang and Takane, 2014). To empirically test the difference, we considered five sample sizes, ranging from what have been previously reported as small (*n* = 50, 100, 200), medium (*n* = 500), and large (*n* = 1000).

The algorithms used for model estimation included ALS estimator for GSCA and ML and WLSMV estimators for CSA. Previous research, including Li (2016), Flora and Curran (2004), and Muthén et al. (1997), has shown that WLSMV is superior to ML in case of ordinal observed variables in terms of accuracy and bias. At the same time, WLSMV is known to have convergence-related stringencies related to low sample size (Flora and Curran, 2004). For this reason, we have chosen to include both estimation methods for CSA.

In sum, there were 2 (misspecification: yes/no) × 4 (number of response categories) × 2 (observed variable distributions) × 5 (sample sizes) = 80 experimental conditions in the current simulation. Five hundred samples were drawn in each of 80 conditions, yielding a total of 40,000 replications. Data generation was completed using the MONTECARLO command in M*plus* 7 (Muthén and Muthén, 1998–2012). Data analysis was performed using M*plus* 7 for CSA models and using the R software (R Core Team, 2017) with the *gesca* package (Hwang et al., 2016) for GSCA models.

### Evaluation Criteria

We considered two primary evaluation criteria for parameter recovery: (a) average relative bias of parameter estimates, (b) average root-mean-squared error of parameter estimates. The formulae for relative bias (RB) and root mean square error (RMSE) for the estimated value $\hat{\theta }$ of each parameter *k* (*k* = 1, …, *p*) in replication *j* (*j* = 1, …, *r*) relative to its respective population parameter value θ, are as follows:

$$RB\left({\hat{\theta }}_{k}\right)=\frac{1}{r}\sum_{j=1}^{r}\left[\frac{{\hat{\theta }}_{kj}-\theta_{k}}{\theta_{k}}\right]\times 100\%$$

and

$$RMSE\left({\hat{\theta }}_{k}\right)=\sqrt{\frac{1}{r}\sum_{j=1}^{r}{\left[\frac{{\hat{\theta }}_{kj}-\theta_{k}}{\theta_{k}}\right]}^{2}.}$$

RB quantifies the amount to which the estimated parameter values detract from the true parameter values. This measure further indicates the degree to which the chosen algorithm properly estimates the model parameters. On the other hand, RMSE represents the degree to which the estimated parameter values vary around the parameter value, thereby incorporating both bias and variance of the estimated parameters. For this study, we calculated means of the RB and RMSE across the loading and path coefficients to ascertain the average value of each measure across each type of parameter:

$$RB_{a}\left(\hat{\theta }\right)=\frac{1}{p}\sum_{k=1}^{p}RB\left({\hat{\theta }}_{k}\right)$$

and

$$RMSE_{a}\left(\hat{\theta }\right)=\frac{1}{p}\sum_{k=1}^{p}RMSE\left({\hat{\theta }}_{k}\right).$$

Although we acknowledge the importance of adhering to a singular criterion for non-ignorable bias in parameter estimates, we agree with Reise et al. (2013) that in many similar cases, parameter bias is largely context dependent and therefore comparing against an absolute criterion can be misleading. We therefore chose to interpret parameter bias as well as error within each study condition and our conclusions were drawn relative to the context.

### Non-convergence

The quality of the results was also evaluated for each condition by considering the proportion of non-converged solutions. We define non-convergence as instance in which either (1) the software program exceeds the number of default iterations without meeting the usual convergence criterion, or (2) the model converged on an improper solution that contains one or more non-positive definite matrices, also referred to as a Heywood case. In either case, the non-converged solutions were excluded from any further analysis and were not incorporated in the presented results.

## Results

### Parameter Recovery

We began by determining the simulation conditions that produced substantial variability in the path coefficients and loadings. In order to conceptualize variability as distinct from the specified fluctuation in the model parameters, we calculated the mean absolute difference (MAD) of parameters and their estimates as follows:

$$MAD=\frac{\sum_{j=1}^{P}\left|{\hat{\theta }}_{j}-\theta_{j}\right|}{P}$$

where ${\hat{\theta }}_{j}$ and θ_*j*_ are an estimate and its parameter, respectively, and P is the number of parameters. The MAD of the estimated parameters ${\hat{\theta }}_{j}$ was analyzed as the outcome in an analysis of variance (ANOVA) model, with the five simulation design parameters serving as predictors. The interaction terms of those design parameters were also included as predictors in up to a maximum-possible 5-way interaction. Due to the large number of available observations, we focused on effect sizes (e.g., η^2^) of the predictors rather than their statistical significance (see Paxton et al., 2001), and decided to interpret only those effects which were at least medium in magnitude, e.g., η^2^ ≥ 0.06 (Cohen, 1988).

For the path coefficients, the estimator (η^2^ = 0.49), model misspecification (η^2^ = 0.18), and observed variable distribution (η^2^ = 0.07) were considered, as were their interactions: estimator by misspecification (η^2^ = 0.56), estimator by distribution (η^2^ = 0.08), and distribution by misspecification (η^2^ = 0.11). For the loadings, only the estimator was considered (η^2^ = 0.84). Note that all the exact values of RMSE and RB of loadings and path coefficients are provided in tables as Supplementary Materials.

### Loadings

**Figure 2:**
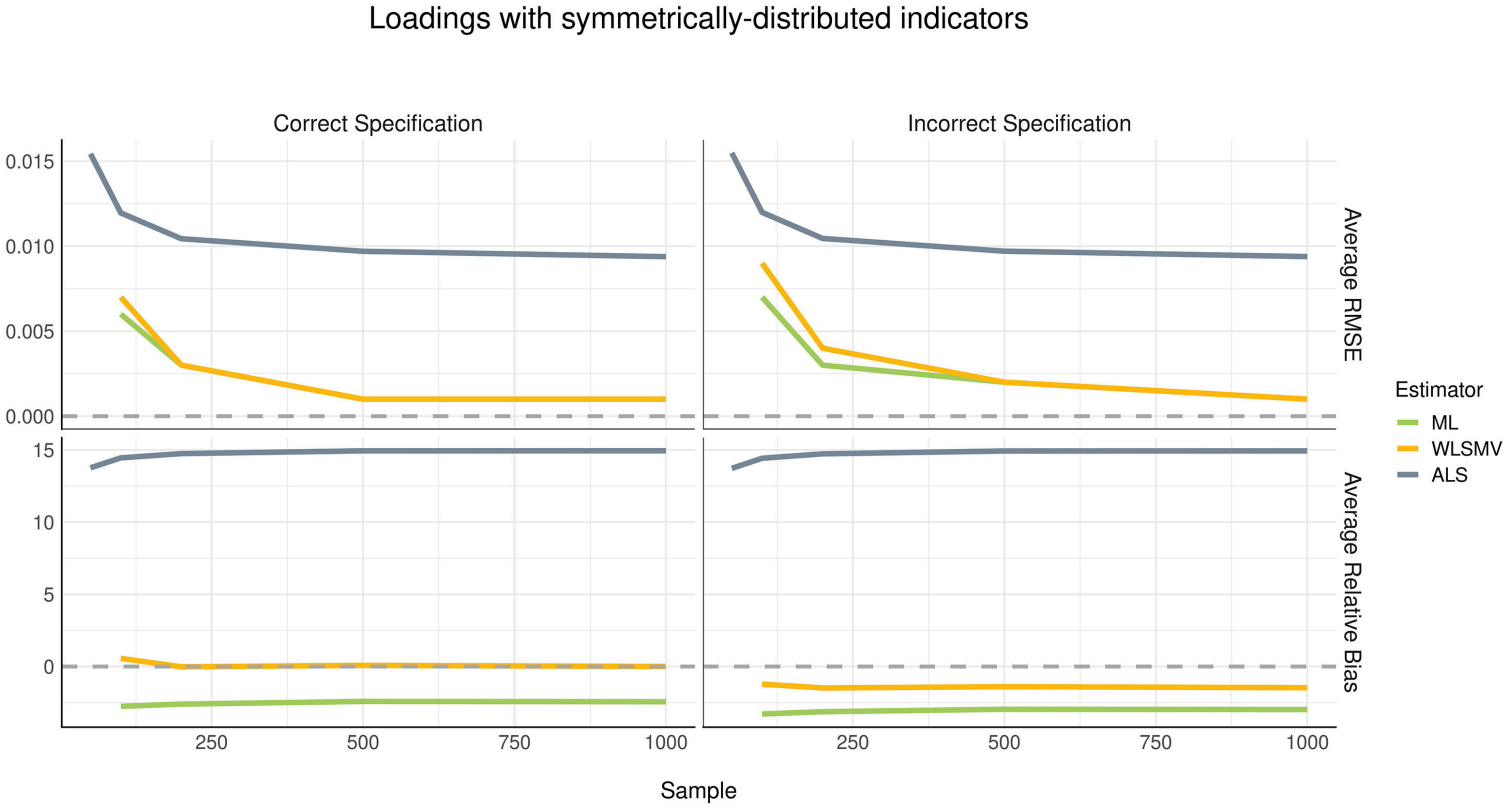
Estimation error and bias in loadings with symmetrically distributed indictors.

**Figure 3:**
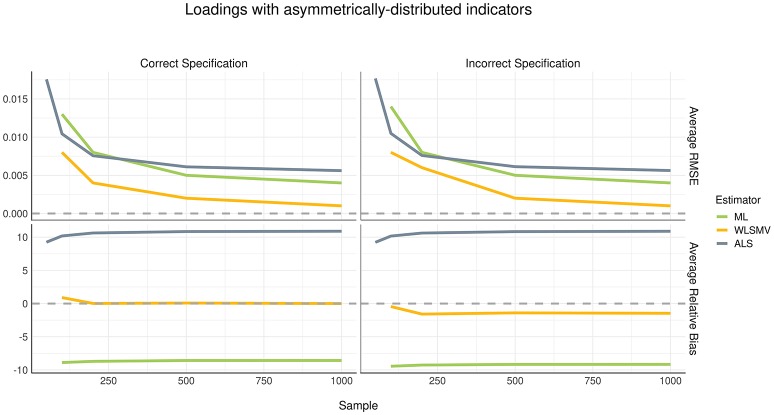
Estimation error and bias in loadings with asymmetrically distributed indictors.

Considering the current simulation incorporated model misspecification at the structural level but not at the measurement level, we did not expect to see a difference in recovery of loading parameters between the correct and incorrect specification conditions. This finding was confirmed, as shown in Figures 2, 3. The pattern of parameter recovery was also similar between the symmetric and asymmetric distribution conditions. That is, in both distribution conditions, average RMSE suggested that CSA-WLSMV (WLSMV, hereafter) had the lowest estimation error, followed by CSA-ML (ML, hereafter) and lastly GSCA-ALS (ALS, hereafter). However, ALS showed similar performance to both CSA conditions when the indicators where asymmetrically distributed. According to average RB, in both indicator-symmetry conditions, ML and WLSMV had better recovery than ALS. Overall, ML was more biased relative to WLSMV but even more so in the asymmetric condition.

For all three algorithms, in general, average RMSE decreased as the sample size increased in a non-linear (quadratic) fashion. However, sample size had no effect on bias—see the relatively flat trajectories of average RB over different sample sizes in the figures.

### Path Coefficients

**Figure 4:**
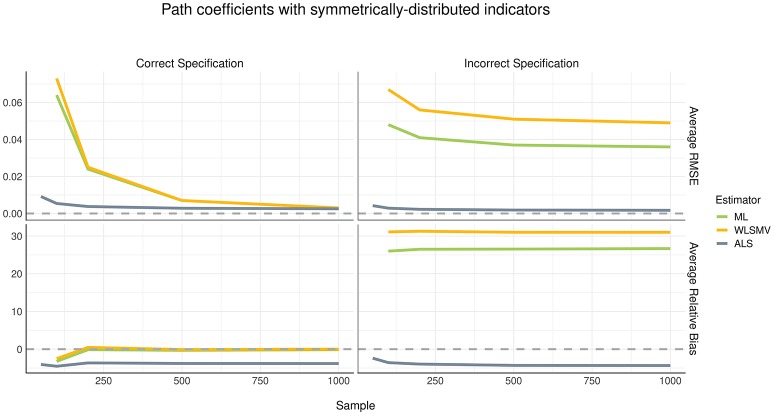
Estimation error and bias in structural path coefficients with symmetrically-distributed indicators.

**Figure 5:**
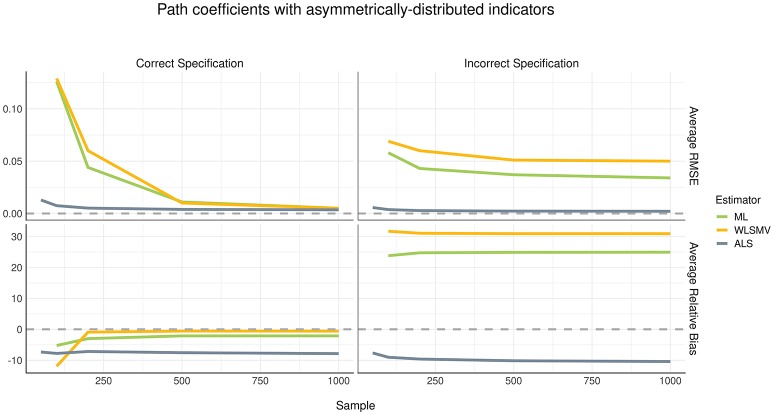
Estimation error and bias in structural path coefficients with asymmetrically-distributed indicators.

Although the pattern of parameter recovery in path coefficients was similar between the symmetric and asymmetric distribution conditions, the effect of model misspecification was compelling, as shown in Figures 4, 5. In the correct specification condition (see left panels of Figures 4, 5), average RMSE suggested that ALS had much lower estimation error than both ML and WLSMV when the sample size was small, but at a sample size of 500 or greater, the amount of estimation error became negligible in each algorithm. According to average RB, when the (structural) model was correctly specified, ML and WLSMV had lower estimation error than ALS. On the other hand, under model misspecification (see right panels of Figures 4, 5), ALS outperformed the CSA-based methods in terms of both average RMSE and average RB, regardless of sample size.

### Non-convergence

The non-convergence issues of CSA-based models resided primarily with small sample sizes, which is to be expected due to the complexity of the population model. Markedly, neither ML nor WLSMV yielded any successfully converged solutions in the conditions with a sample size of 50. The two CSA-based estimation methods experienced similar non-convergence problems in the other small sample size conditions—the most problematic conditions were those in which the model contained asymmetrically-distributed observed variables with more than four categories, a sample size of 100, and was estimated with WLSMV (41–57% converged). A lesser degree of non-convergence was observed in the model that was also estimated with WLSMV but had either asymmetrically-distributed variables with four categories or symmetrically-distributed variables with any number of categories (73–79% converged). The least severe conditions for non-convergence involved asymmetric distribution, <6 categories, a sample size of 100, and ML (85–94% converged). All other conditions at a sample size of 200 and below had trivial amount of non-convergence (95–99% converged). No non-convergence was observed for CSA-based models with a sample size of 500 or greater. Under ALS estimation, there were no instances of non-convergence even at the smallest sample size of 50, suggesting that ALS vastly outperformed both ML and WLSMV on this metric.

## Discussion

In this Monte Carlo simulation study, we demonstrated the relative performance of GSCA-ALS, CSA-ML, and CSA-WLSMV with ordinal observed variables in terms of parameter recovery. The major findings can be summarized as follows. First, when the structural model was correctly specified, all three algorithms under-estimated the parameter values of path coefficients in a similar degree when the sample size was small. However, both CSA-ML and CSA-WLSMV produced unbiased path coefficient estimates when the sample size was 200 or larger, compared to GSCA-ALS. On the other hand, taking into account both bias and variability of the parameter estimates (i.e., average RMSE), GSCA-ALS showed much smaller error in estimating path coefficients than both CSA-ML and CSA-WLSMV when the sample size was small, but at a sample size of 500 or larger the magnitude of estimation error became negligible across all three algorithms. Second, when the structural model was incorrectly specified, GSCA-ALS outperformed the two CSA-based methods in terms of parameter recovery of the path coefficients. That is, GSCA-ALS showed a trivial amount of estimation error compared to both CSA-ML and CSA-WLSMV and overwhelmingly smaller biases, regardless of the sample size. Third, the different conditions of observed variable distribution (i.e., symmetric or asymmetric) did not lead to considerable differences in parameter recovery of the path coefficients among the methods. On the other hand, the simulation results for the loadings indicated that under the asymmetric distribution condition, GSCA-ALS had lower estimation error than CSA-ML and CSA-WLSMV, and both CSA-ML and GSCA-ALS produced extremely biased loading estimates. In the symmetric distribution condition, both CSA-ML and CSA-WLSMV generally had better recovery of the loadings than GSCA-ALS in terms of both estimation error and bias. Fourth, CSA-ML and CSA-WLSMV suffered from massive-to-moderate non-convergence problems with small sample sizes (i.e., 50, 100, and 200). On the other hand, GSCA-ALS did not encounter any convergence problems even at the smallest sample size of 50.

These findings have important implications for researchers in substantive areas who apply structural equation modeling for their non-normally distributed ordered data. The choice over different estimation algorithms should be carefully considered especially when the sample size is less than moderate or/and when they are uncertain if the model has been correctly specified. Our stimulation results revealed outperformance of GSCA-ALS over both CSA-ML and CSA-WLSMV under model misspecification. Thus, we would recommend the adoption of GSCA-ALS when a correct specification of (structural) model cannot be ensured. In addition, when the sample size is relatively small (e.g., 100 or smaller), our findings suggest GSCS-ALS as the method of choice. In all other circumstances, when the researcher assures the model of being correctly specified, we recommend CSA-WLSMV. The WLSMV estimator has demonstrated superior parameter recovery for both loadings and path coefficients, when the model is correctly specified.

Despite these significant contributions, the current study has several limitations. Most apparently, for the comparative study of GSCA and CSA in parameter recovery, we generated simulation data within the CSA framework—i.e., assuming that latent variables are approximated by common factors rather than components of observed variables. The measurement model in CSA is typically a confirmatory factor-analytic model, where common factors explain the covariances among indicators and unique factors represent measurement error. On the other hand, the measurement model in GSCA is a confirmatory component-analytic model (e.g., Kiers et al., 1996), where components aim to explain the entire variances of the indicators with no distinction between common and unique variances. In this sense, GSCA cannot define and handle measurement error in the same way as common factor analysis, tending to produce biased parameter estimates of factor-based models. Consequently, GSCA might be placed at a disadvantage in this simulation study.

We should have considered alternative data generation procedures as well in a fair manner. Moreover, although the present simulation involved a more realistic structural model and most relevant conditions, the relative performance of each method might be conditional on the specific levels chosen for the experimental conditions. Thus, it might be necessary to consider a broad range of conditions and models for more rigorous investigations.

Given the superiority of GSCA-ALS over CSA-ML and CSA-WLSMV under model misspecification, future studies would expand their scope to other robust CSA methods for a comparison with GSCA-ALS. For instance, Bollen (1996) recommended using two-stage least squares (2SLS) estimation for CSA in the presence of incorrectly specified models. CSA-2SLS is a limited-information estimation procedure that employs one equation at a time in parameter estimation, and consequently a specification error in one equation does not necessarily affects the other equations. Bollen et al. (2007) have empirically demonstrated the robustness of 2SLS to misspecification, as compared to CSA-ML. Thus, a Monte Carlo simulation on the relative performance of CSA-2SLS and GSCA-ALS under various misspecification conditions would ascertain the relative benefits of each approach. Another direction for future studies is to compare GSCA-ALS with its regularized extension (rGSCA-ALS; (Hwang, 2009)). rGSCA-ALS combines a ridge type of regularization into GSCA in a unified framework, thereby handling potential multicollinearity problems more effectively. In the preliminary simulation study, rGSCA-ALS was found to provide parameter estimates that are as good as or better than those from original GSCA-ALS in various conditions of normally-distributed data. Therefore, it is suggested to compare GSCA-ALS and rGSCA-ALS under more realistic situations involving observed ordinal variables and the presence of multicollinearity.

## Author Contributions

KJ contributed to technical development, empirical analyses, and manuscript writing. PP contributed to technical development, empirical analyses, and manuscript writing. JL contributed to manuscript writing, and HH contributed to manuscript writing.

### Conflict of Interest Statement

The authors declare that the research was conducted in the absence of any commercial or financial relationships that could be construed as a potential conflict of interest.
